# Ethyl 2-{3-[(6-chloro­pyridin-3-yl)meth­yl]-2-(nitro­imino)­imidazolidin-1-yl}acetate

**DOI:** 10.1107/S160053681200918X

**Published:** 2012-03-07

**Authors:** Kamini Kapoor, Madhukar B. Deshmukh, Chetan S. Shripanavar, Vivek K. Gupta, Rajni Kant

**Affiliations:** a X-ray Crystallography Laboratory, Post-Graduate Department of Physics & Electronics, University of Jammu, Jammu Tawi 180 006, India; b Department of Chemistry, Shivaji University, Kolhapur, 416 004, India

## Abstract

In the title compound, C_13_H_16_ClN_5_O_4_, the imidazole ring is in a slight envelope conformation. The dihedral angle between the pyridine ring and the four essentially planar atoms [maximum deviation 0.015 (2) Å] of the imidazole ring is 80.8 (1)°. In, the crystal, weak C—H⋯O and C—H⋯N hydrogen bonds are present. In addition, there are weak π–π stacking inter­actions between symmetry-related pyridine rings with a centroid–centroid distance of 3.807 (1) Å.

## Related literature

For background to the insecticidal applications of imidacloprid [systematic name: (*E*)-1-(6-chloro-3-pyridyl­meth­yl)-*N*-nitro­imidazolidin-2-yl­idene­amine], see: Deshmukh *et al.* (2011 ▶, 2012 ▶); Zhao *et al.* (2010 ▶). For related structures, see: Kapoor *et al.* (2011 ▶, 2012 ▶); Kant *et al.* (2012 ▶).
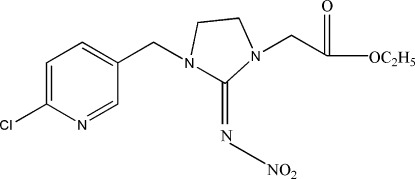


## Experimental

### 

#### Crystal data

C_13_H_16_ClN_5_O_4_
*M* *_r_* = 341.76
Monoclinic, 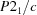

*a* = 7.8136 (2) Å
*b* = 19.3483 (4) Å
*c* = 10.1926 (2) Å
β = 100.346 (2)°
*V* = 1515.86 (6) Å^3^
*Z* = 4
Mo *K*α radiation
μ = 0.28 mm^−1^
*T* = 293 K
0.3 × 0.2 × 0.1 mm

#### Data collection

Oxford Diffraction Xcalibur Sapphire3 diffractometer
Absorption correction: multi-scan (*CrysAlis PRO RED*; Oxford Diffraction, 2010 ▶) *T*_min_ = 0.868, *T*_max_ = 1.000
47458 measured reflections
2983 independent reflections
2387 reflections with *I* > 2σ(*I*)
*R*_int_ = 0.046

#### Refinement

*R*[*F* ^2^ > 2σ(*F* ^2^)] = 0.038
*wR*(*F* ^2^) = 0.090
*S* = 1.02
2983 reflections
209 parameters
H-atom parameters constrained
Δρ_max_ = 0.23 e Å^−3^
Δρ_min_ = −0.29 e Å^−3^

### 

Data collection: *CrysAlis PRO CCD* (Oxford Diffraction, 2010 ▶); cell refinement: *CrysAlis PRO CCD*; data reduction: *CrysAlis PRO RED* (Oxford Diffraction, 2010 ▶); program(s) used to solve structure: *SHELXS97* (Sheldrick, 2008 ▶); program(s) used to refine structure: *SHELXL97* (Sheldrick, 2008 ▶); molecular graphics: *ORTEP-3* (Farrugia, 1997 ▶) and *PLATON* (Spek, 2009 ▶); software used to prepare material for publication: *PLATON*.

## Supplementary Material

Crystal structure: contains datablock(s) I, global. DOI: 10.1107/S160053681200918X/lh5425sup1.cif

Structure factors: contains datablock(s) I. DOI: 10.1107/S160053681200918X/lh5425Isup2.hkl

Supplementary material file. DOI: 10.1107/S160053681200918X/lh5425Isup3.cml

Additional supplementary materials:  crystallographic information; 3D view; checkCIF report

## Figures and Tables

**Table 1:** Hydrogen-bond geometry (Å, °)

*D*—H⋯*A*	*D*—H	H⋯*A*	*D*⋯*A*	*D*—H⋯*A*
C9—H9*B*⋯O15^i^	0.97	2.50	3.358 (2)	147
C17—H17*A*⋯N1^ii^	0.97	2.57	3.509 (2)	163

Symmetry codes: (i) 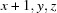; (ii) 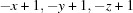.

**Fig. 1.**
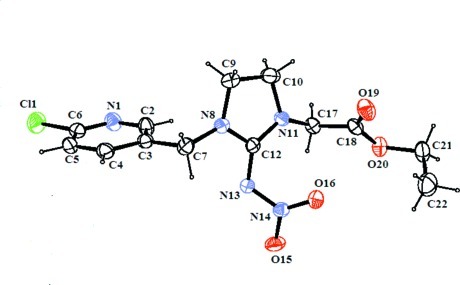
The molecular structure with ellipsoids are drawn at the 40% probability level. H atoms are shown as small spheres of arbitrary radii.

**Fig. 2.**
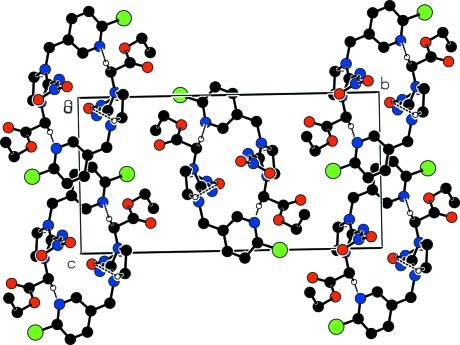
Part of the crystal structure with weak hydrogen bonds shown as dashed lines.

## supplementary crystallographic information

### Comment

The discovery of imidacloprid has been referred to as a milestone in the past three decades of insecticidal research. The nitroguanidine moiety of imidacloprid is also a common site for metabolism *via* cleavage to the guanidine and reduction to di-nitro-imidacloprid. The insecticidal activity of nitroguanidine was found to be 10,000 fold higher than that of natural insecticide nicotine (Deshmukh *et al.*, 2012). In mammalian systems the nitro group of imidacloprid has been postulated to be reduced to nitrosoguanidine and aminoguanidine and then cleaved to the guanidine and urea derivatives (Deshmukh *et al.*, 2011). Therefore, in a search for new neonicotinoid insecticides with improved profiles, neonicotinoid derivatives containing N-oxalyl groups were designed and synthesized (Zhao *et al.*, 2010).

The molecular structure of the title compound is shown in Fig. 1. The bond lengths and angles are comparable to those common to related structures (Kapoor *et al.*, 2011,2012; Kant *et al.*, 2012). The imidazole ring is in a slight envelope conformation with atom C9 forming the flap. The dihedral angle between the pyridine ring [N1/C2-C6] and the four essentially planar atoms [N8/N11/C10/C12 (maximum deviation 0.015 (2)Å for C12)] of the imidazole ring is 80.8 (1)°. In the crystal, molecules are connected by pairs of weak C—H···O hydrogen bonds into centrosymmetric dimers, which are in turn, linked into columns along [100] by weak C—H···N hydrogen bonds (Fig .2). In addition, there is a weak π···π interaction between the pyridine ring at (*x*, *y*, *z*) and the pyridine ring at (1 - *x*, 1 - *y*, - *z*) [centroid separation = 3.807 (1) Å, interplanar spacing = 3.368 Å and centroid shift = 1.77 Å].

### Experimental

Imidacloprid (10.20 g, 0.04 mol) in 30 ml acetone, ethyl chloroacetate (7.32 g, 0.06 mol) was refluxed for about 24 h in presence of 10 g m K_2_CO_3_. An aliquot of sample was taken to monitor the progress of reaction by TLC. After completion of reaction, the hot reaction mixture was filtered to remove excess K_2_CO_3_. Filtrate was then dried under reduced pressure giving a white solid, Yield 80%. The synthesized compound was dissolved in methanol, by the process of slow evaporation a fine crystalline compound separated out.

### Refinement

H atoms were positioned geometrically and were treated as riding on their parent C atoms, with C—H distances of 0.93–0.97 Å.

### Crystal data

**Table:**

C_13_H_16_ClN_5_O_4_	*F*(000) = 712
*M**_r_* = 341.76	*D*_x_ = 1.498 Mg m^−^^3^
Monoclinic, *P*2_1_/*c*	Mo *K*α radiation, λ = 0.71073 Å
Hall symbol: -P 2ybc	Cell parameters from 21411 reflections
*a* = 7.8136 (2) Å	θ = 3.6–29.1°
*b* = 19.3483 (4) Å	µ = 0.28 mm^−^^1^
*c* = 10.1926 (2) Å	*T* = 293 K
β = 100.346 (2)°	Plate, white
*V* = 1515.86 (6) Å^3^	0.3 × 0.2 × 0.1 mm
*Z* = 4	

### Data collection

**Table:**

Oxford Diffraction Xcalibur Sapphire3 diffractometer	2983 independent reflections
Radiation source: fine-focus sealed tube	2387 reflections with *I* > 2σ(*I*)
Graphite monochromator	*R*_int_ = 0.046
Detector resolution: 16.1049 pixels mm^-1^	θ_max_ = 26.0°, θ_min_ = 3.7°
ω scans	*h* = −9→9
Absorption correction: multi-scan (*CrysAlis PRO* RED; Oxford Diffraction, 2010)	*k* = −23→23
*T*_min_ = 0.868, *T*_max_ = 1.000	*l* = −12→12
47458 measured reflections	

### Refinement

**Table:**

Refinement on *F*^2^	Primary atom site location: structure-invariant direct methods
Least-squares matrix: full	Secondary atom site location: difference Fourier map
*R*[*F*^2^ > 2σ(*F*^2^)] = 0.038	Hydrogen site location: inferred from neighbouring sites
*wR*(*F*^2^) = 0.090	H-atom parameters constrained
*S* = 1.02	*w* = 1/[σ^2^(*F*_o_^2^) + (0.0372*P*)^2^ + 0.6841*P*] where *P* = (*F*_o_^2^ + 2*F*_c_^2^)/3
2983 reflections	(Δ/σ)_max_ = 0.001
209 parameters	Δρ_max_ = 0.23 e Å^−^^3^
0 restraints	Δρ_min_ = −0.29 e Å^−^^3^

### Special details

**Table:**

Experimental. *CrysAlis PRO*, Oxford Diffraction Ltd., Version 1.171.34.40 (release 27–08-2010 CrysAlis171. NET) (compiled Aug 27 2010,11:50:40) Empirical absorption correction using spherical harmonics, implemented in SCALE3 ABSPACK scaling algorithm.IR (cm-1): 3008, 2991, 2908, 1743, 1558, 1548. 1H NMR ?: 1.29(t, J: 7.5 Hz, CH3),3.59(t, J: 7.5 Hz, CH2), 3.84(t, J: 7.5 Hz, CH2), 4.06 (s, CH2), 4.24 (q, J:7.5 Hz, OCH2), 4.50 (s, CH2), 7.37 (d, J: 8.2 Hz, Py1H), 7.74 (dd, J1: 7.5,J2: 2.5 Hz, Py1H), 8.32 (s, Py1H) p.p.m.. LCMS/MS (ESI, m/*z*): 342.0891 (*M*+H)+,295.0881, 261.1297, 170.0910.
Geometry. All e.s.d.'s (except the e.s.d. in the dihedral angle between two l.s. planes) are estimated using the full covariance matrix. The cell e.s.d.'s are taken into account individually in the estimation of e.s.d.'s in distances, angles and torsion angles; correlations between e.s.d.'s in cell parameters are only used when they are defined by crystal symmetry. An approximate (isotropic) treatment of cell e.s.d.'s is used for estimating e.s.d.'s involving l.s. planes.
Refinement. Refinement of *F*^2^ against ALL reflections. The weighted *R*-factor *wR* and goodness of fit *S* are based on *F*^2^, conventional *R*-factors *R* are based on *F*, with *F* set to zero for negative *F*^2^. The threshold expression of *F*^2^ > σ(*F*^2^) is used only for calculating *R*-factors(gt) *etc*. and is not relevant to the choice of reflections for refinement. *R*-factors based on *F*^2^ are statistically about twice as large as those based on *F*, and *R*- factors based on ALL data will be even larger.

### Fractional atomic coordinates and isotropic or equivalent isotropic displacement parameters (Å^2^)

**Table:**

	*x*	*y*	*z*	*U*_iso_*/*U*_eq_	
Cl1	0.49235 (8)	0.34128 (3)	−0.00544 (6)	0.06124 (18)	
N1	0.4056 (2)	0.42363 (8)	0.17333 (16)	0.0437 (4)	
C2	0.3295 (2)	0.48018 (10)	0.21371 (18)	0.0402 (4)	
H2	0.3394	0.4877	0.3049	0.048*	
C3	0.2376 (2)	0.52773 (9)	0.12776 (16)	0.0316 (4)	
C4	0.2223 (2)	0.51489 (10)	−0.00787 (17)	0.0376 (4)	
H4	0.1600	0.5453	−0.0692	0.045*	
C5	0.2988 (2)	0.45741 (10)	−0.05220 (18)	0.0406 (4)	
H5	0.2903	0.4481	−0.1426	0.049*	
C6	0.3884 (2)	0.41447 (10)	0.04397 (19)	0.0387 (4)	
C7	0.1554 (2)	0.59159 (10)	0.17545 (16)	0.0362 (4)	
H7A	0.1805	0.6309	0.1230	0.043*	
H7B	0.0302	0.5855	0.1602	0.043*	
N8	0.21587 (18)	0.60656 (8)	0.31527 (14)	0.0337 (3)	
C9	0.3777 (2)	0.64367 (10)	0.36251 (18)	0.0377 (4)	
H9A	0.3764	0.6890	0.3218	0.045*	
H9B	0.4774	0.6180	0.3440	0.045*	
C10	0.3805 (2)	0.64912 (11)	0.5113 (2)	0.0459 (5)	
H10A	0.4723	0.6208	0.5610	0.055*	
H10B	0.3969	0.6966	0.5414	0.055*	
N11	0.20831 (18)	0.62338 (7)	0.52662 (14)	0.0314 (3)	
C12	0.1228 (2)	0.59805 (8)	0.41189 (16)	0.0278 (3)	
N13	−0.02640 (17)	0.56113 (7)	0.38209 (14)	0.0324 (3)	
N14	−0.15702 (18)	0.57746 (7)	0.44638 (14)	0.0330 (3)	
O15	−0.28030 (16)	0.53623 (7)	0.43068 (15)	0.0490 (4)	
O16	−0.15984 (16)	0.63170 (7)	0.51017 (13)	0.0454 (3)	
C17	0.1705 (2)	0.61047 (9)	0.65820 (16)	0.0331 (4)	
H17A	0.2751	0.5946	0.7165	0.040*	
H17B	0.0836	0.5743	0.6531	0.040*	
C18	0.1042 (2)	0.67502 (9)	0.71615 (17)	0.0347 (4)	
O19	0.13493 (19)	0.73282 (7)	0.68446 (14)	0.0505 (4)	
O20	0.01369 (17)	0.65861 (6)	0.81063 (12)	0.0414 (3)	
C21	−0.0639 (3)	0.71622 (11)	0.8720 (2)	0.0502 (5)	
H21A	0.0215	0.7527	0.8939	0.060*	
H21B	−0.0980	0.7007	0.9541	0.060*	
C22	−0.2189 (3)	0.74367 (13)	0.7804 (3)	0.0658 (7)	
H22A	−0.1828	0.7651	0.7049	0.099*	
H22B	−0.2765	0.7772	0.8266	0.099*	
H22C	−0.2975	0.7064	0.7505	0.099*	

### Atomic displacement parameters (Å^2^)

**Table:**

	*U*^11^	*U*^22^	*U*^33^	*U*^12^	*U*^13^	*U*^23^
Cl1	0.0666 (4)	0.0457 (3)	0.0760 (4)	0.0005 (3)	0.0250 (3)	−0.0183 (3)
N1	0.0488 (10)	0.0409 (9)	0.0396 (9)	0.0051 (7)	0.0028 (7)	−0.0019 (7)
C2	0.0482 (11)	0.0440 (11)	0.0273 (9)	0.0050 (9)	0.0036 (8)	−0.0007 (8)
C3	0.0283 (9)	0.0374 (10)	0.0288 (9)	−0.0033 (7)	0.0048 (7)	0.0013 (7)
C4	0.0338 (10)	0.0483 (11)	0.0296 (9)	−0.0034 (8)	0.0025 (7)	0.0026 (8)
C5	0.0402 (10)	0.0523 (12)	0.0296 (9)	−0.0105 (9)	0.0071 (8)	−0.0089 (8)
C6	0.0335 (9)	0.0387 (10)	0.0447 (11)	−0.0064 (8)	0.0089 (8)	−0.0093 (8)
C7	0.0343 (9)	0.0445 (10)	0.0296 (9)	0.0042 (8)	0.0056 (7)	0.0040 (8)
N8	0.0303 (7)	0.0393 (8)	0.0320 (8)	−0.0024 (6)	0.0065 (6)	−0.0023 (6)
C9	0.0274 (9)	0.0414 (10)	0.0443 (10)	−0.0021 (8)	0.0063 (8)	0.0026 (8)
C10	0.0345 (10)	0.0563 (13)	0.0459 (11)	−0.0121 (9)	0.0043 (8)	−0.0037 (9)
N11	0.0316 (7)	0.0311 (8)	0.0309 (7)	−0.0033 (6)	0.0042 (6)	−0.0024 (6)
C12	0.0297 (9)	0.0224 (8)	0.0310 (9)	0.0045 (6)	0.0048 (7)	0.0003 (6)
N13	0.0293 (7)	0.0340 (8)	0.0346 (8)	−0.0030 (6)	0.0074 (6)	−0.0059 (6)
N14	0.0296 (8)	0.0326 (8)	0.0355 (8)	0.0027 (6)	0.0026 (6)	0.0025 (6)
O15	0.0304 (7)	0.0429 (8)	0.0743 (10)	−0.0071 (6)	0.0114 (6)	−0.0031 (7)
O16	0.0414 (7)	0.0428 (8)	0.0529 (8)	0.0053 (6)	0.0104 (6)	−0.0131 (6)
C17	0.0380 (10)	0.0303 (9)	0.0293 (9)	0.0014 (7)	0.0014 (7)	0.0010 (7)
C18	0.0392 (10)	0.0339 (10)	0.0282 (9)	−0.0018 (8)	−0.0013 (7)	−0.0026 (7)
O19	0.0711 (10)	0.0294 (7)	0.0525 (8)	−0.0076 (7)	0.0151 (7)	−0.0041 (6)
O20	0.0550 (8)	0.0366 (7)	0.0345 (7)	0.0050 (6)	0.0127 (6)	−0.0010 (5)
C21	0.0605 (13)	0.0481 (12)	0.0431 (11)	0.0052 (10)	0.0125 (10)	−0.0134 (9)
C22	0.0676 (15)	0.0568 (15)	0.0716 (16)	0.0174 (12)	0.0091 (12)	−0.0097 (12)

### Geometric parameters (Å, º)

**Table:**

Cl1—C6	1.7510 (19)	C10—H10A	0.9700
N1—C6	1.313 (2)	C10—H10B	0.9700
N1—C2	1.345 (2)	N11—C12	1.332 (2)
C2—C3	1.380 (2)	N11—C17	1.446 (2)
C2—H2	0.9300	C12—N13	1.354 (2)
C3—C4	1.388 (2)	N13—N14	1.3458 (19)
C3—C7	1.512 (2)	N14—O16	1.2367 (19)
C4—C5	1.377 (3)	N14—O15	1.2388 (18)
C4—H4	0.9300	C17—C18	1.512 (2)
C5—C6	1.376 (3)	C17—H17A	0.9700
C5—H5	0.9300	C17—H17B	0.9700
C7—N8	1.448 (2)	C18—O19	1.200 (2)
C7—H7A	0.9700	C18—O20	1.332 (2)
C7—H7B	0.9700	O20—C21	1.462 (2)
N8—C12	1.335 (2)	C21—C22	1.488 (3)
N8—C9	1.459 (2)	C21—H21A	0.9700
C9—C10	1.517 (3)	C21—H21B	0.9700
C9—H9A	0.9700	C22—H22A	0.9600
C9—H9B	0.9700	C22—H22B	0.9600
C10—N11	1.469 (2)	C22—H22C	0.9600
			
C6—N1—C2	116.47 (16)	C9—C10—H10B	111.1
N1—C2—C3	123.83 (16)	H10A—C10—H10B	109.0
N1—C2—H2	118.1	C12—N11—C17	126.65 (14)
C3—C2—H2	118.1	C12—N11—C10	110.86 (14)
C2—C3—C4	117.05 (16)	C17—N11—C10	120.06 (14)
C2—C3—C7	122.91 (15)	N11—C12—N8	110.38 (14)
C4—C3—C7	120.04 (15)	N11—C12—N13	131.84 (15)
C5—C4—C3	120.43 (17)	N8—C12—N13	117.42 (14)
C5—C4—H4	119.8	N14—N13—C12	117.67 (13)
C3—C4—H4	119.8	O16—N14—O15	121.84 (14)
C6—C5—C4	116.69 (16)	O16—N14—N13	122.81 (14)
C6—C5—H5	121.7	O15—N14—N13	115.21 (14)
C4—C5—H5	121.7	N11—C17—C18	111.17 (14)
N1—C6—C5	125.52 (17)	N11—C17—H17A	109.4
N1—C6—Cl1	115.38 (15)	C18—C17—H17A	109.4
C5—C6—Cl1	119.10 (14)	N11—C17—H17B	109.4
N8—C7—C3	113.43 (14)	C18—C17—H17B	109.4
N8—C7—H7A	108.9	H17A—C17—H17B	108.0
C3—C7—H7A	108.9	O19—C18—O20	125.08 (17)
N8—C7—H7B	108.9	O19—C18—C17	124.43 (17)
C3—C7—H7B	108.9	O20—C18—C17	110.43 (15)
H7A—C7—H7B	107.7	C18—O20—C21	116.27 (15)
C12—N8—C7	125.22 (14)	O20—C21—C22	110.91 (16)
C12—N8—C9	111.84 (14)	O20—C21—H21A	109.5
C7—N8—C9	122.24 (14)	C22—C21—H21A	109.5
N8—C9—C10	102.70 (14)	O20—C21—H21B	109.5
N8—C9—H9A	111.2	C22—C21—H21B	109.5
C10—C9—H9A	111.2	H21A—C21—H21B	108.0
N8—C9—H9B	111.2	C21—C22—H22A	109.5
C10—C9—H9B	111.2	C21—C22—H22B	109.5
H9A—C9—H9B	109.1	H22A—C22—H22B	109.5
N11—C10—C9	103.54 (14)	C21—C22—H22C	109.5
N11—C10—H10A	111.1	H22A—C22—H22C	109.5
C9—C10—H10A	111.1	H22B—C22—H22C	109.5
N11—C10—H10B	111.1		
			
C6—N1—C2—C3	−0.3 (3)	C17—N11—C12—N8	−165.04 (15)
N1—C2—C3—C4	0.9 (3)	C10—N11—C12—N8	−2.9 (2)
N1—C2—C3—C7	−179.01 (17)	C17—N11—C12—N13	7.7 (3)
C2—C3—C4—C5	−0.9 (3)	C10—N11—C12—N13	169.83 (18)
C7—C3—C4—C5	179.07 (16)	C7—N8—C12—N11	−173.32 (15)
C3—C4—C5—C6	0.3 (3)	C9—N8—C12—N11	−2.8 (2)
C2—N1—C6—C5	−0.4 (3)	C7—N8—C12—N13	12.8 (2)
C2—N1—C6—Cl1	178.84 (13)	C9—N8—C12—N13	−176.68 (14)
C4—C5—C6—N1	0.5 (3)	N11—C12—N13—N14	38.2 (3)
C4—C5—C6—Cl1	−178.79 (13)	N8—C12—N13—N14	−149.51 (15)
C2—C3—C7—N8	13.8 (2)	C12—N13—N14—O16	14.3 (2)
C4—C3—C7—N8	−166.19 (15)	C12—N13—N14—O15	−169.90 (15)
C3—C7—N8—C12	−107.54 (18)	C12—N11—C17—C18	−111.85 (18)
C3—C7—N8—C9	82.8 (2)	C10—N11—C17—C18	87.50 (19)
C12—N8—C9—C10	6.9 (2)	N11—C17—C18—O19	−24.7 (2)
C7—N8—C9—C10	177.74 (16)	N11—C17—C18—O20	157.88 (14)
N8—C9—C10—N11	−7.84 (19)	O19—C18—O20—C21	4.8 (3)
C9—C10—N11—C12	7.0 (2)	C17—C18—O20—C21	−177.77 (14)
C9—C10—N11—C17	170.43 (15)	C18—O20—C21—C22	74.9 (2)

### Hydrogen-bond geometry (Å, º)

**Table:**

*D*—H···*A*	*D*—H	H···*A*	*D*···*A*	*D*—H···*A*
C9—H9*B*···O15^i^	0.97	2.50	3.358 (2)	147
C17—H17*A*···N1^ii^	0.97	2.57	3.509 (2)	163

Symmetry codes: (i) *x*+1, *y*, *z*; (ii) −*x*+1, −*y*+1, −*z*+1.
